# Estimated Under-Five Deaths Associated with Poor-Quality Antimalarials in Sub-Saharan Africa

**DOI:** 10.4269/ajtmh.14-0725

**Published:** 2015-06-03

**Authors:** John P. Renschler, Kelsey M. Walters, Paul N. Newton, Ramanan Laxminarayan

**Affiliations:** Center for Disease Dynamics, Economics and Policy, Washington, District of Columbia; Worldwide Antimalarial Resistance Network, Centre for Tropical Medicine, Churchill Hospital, University of Oxford, United Kingdom; LOMWRU, Microbiology Laboratory, Mahosot Hospital, Vientiane, Lao People's Democratic Republic; Princeton Environmental Institute, Princeton University, Princeton, New Jersey; Public Health Foundation of India, New Delhi, India

## Abstract

Many antimalarials sold in sub-Saharan Africa are poor-quality (falsified, substandard, or degraded), and the burden of disease caused by this problem is inadequately quantified. In this article, we estimate the number of under-five deaths caused by ineffective treatment of malaria associated with consumption of poor-quality antimalarials in 39 sub-Saharan countries. Using Latin hypercube sampling our estimates were calculated as the product of the number of private sector antimalarials consumed by malaria-positive children in 2013; the proportion of private sector antimalarials consumed that were of poor-quality; and the case fatality rate (CFR) of under-five malaria-positive children who did not receive appropriate treatment. An estimated 122,350 (interquartile range [IQR]: 91,577–154,736) under-five malaria deaths were associated with consumption of poor-quality antimalarials, representing 3.75% (IQR: 2.81–4.75%) of all under-five deaths in our sample of 39 countries. There is considerable uncertainty surrounding our results because of gaps in data on case fatality rates and prevalence of poor-quality antimalarials. Our analysis highlights the need for further investigation into the distribution of poor-quality antimalarials and the need for stronger surveillance and regulatory efforts to prevent the sale of poor-quality antimalarials.

## Introduction

Each year malaria causes an estimated 207 million (M) clinical cases worldwide resulting in an estimated 627,000–1,238,000 deaths (0.3–0.6% of clinical cases), the majority in sub-Saharan Africa.^1^,^2^ Children under 5 years of age in this region have the highest risk of contracting and dying from malaria.^3^ Artemisinin-based combination therapies (ACTs) are the first-line treatment recommended by the World Health Organization (WHO) and are vital to reduce the burden of childhood malaria.^1^,^4^,^5^ In Africa, the widespread availability of ACTs through both public and private sectors has lowered malaria morbidity and mortality rates^1^ and reduced the selection pressure for emergence of drug-resistant parasite strains caused by monotherapies.^4^ Despite the clinical advantage of ACTs, many non-artemisinin-based monotherapies (including chloroquine, quinine, halofantrine, and amodiaquine) are still widely available throughout the private sector.^6^,^7^

Many antimalarials sold worldwide are poor-quality. Quality issues span all classes of antimalarial agents including diverse examples of falsification and substandard production (Panel).^3^,^8^ Results aggregated from surveys of antimalarial quality taken between 2001 and 2010 from 21 countries in sub-Saharan Africa show that 35% of samples (796 of 2,297) failed chemical analysis.^3^ Although many antimalarials purchased in sub-Saharan Africa are of poor quality, the quality varies greatly by location.^3^,^9^ A recent review of the Worldwide Antimalarial Resistance Network (WWARN) antimalarial quality database warned against generalizations, given the frequent use of inadequate sampling techniques, the need for standardization of chemical analysis, and the scarcity of samples compared with the total volume of antimalarials consumed.^10^ The review found no reports of antimalarial quality for 17 of the 44 malaria-endemic sub-Saharan African countries.^10^

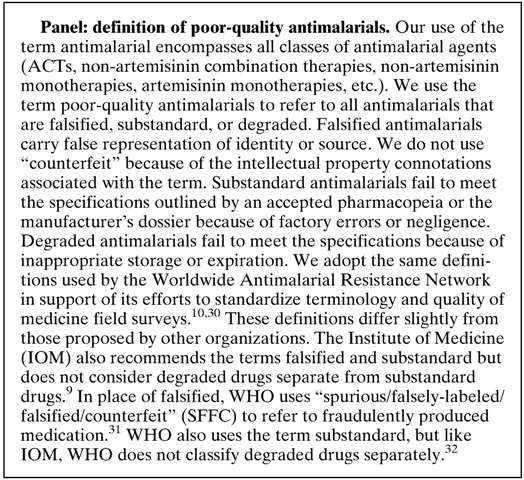

Factors associated with the production and sale of poor-quality antimalarials include inaccessibility and high price of quality ACTs, limited regulatory oversight, lack of penalties, self-prescribing practices, poor knowledge about product authenticity, and a large, unregulated private sector for purchasing pharmaceuticals.^5^,^11^,^12^ The World Health Organization (WHO) estimated that 30% of countries lack any capacity to oversee medicine manufacture, importation, or distribution.^12^ To date, South Africa, Kenya, and Tanzania are the only malaria-endemic African countries with WHO-prequalified laboratories for drug quality testing.^10^

Understanding the market penetration and consequences of poor-quality antimalarials is important but challenging for two reasons. First, many children with malaria are treated in informal private sectors, making it difficult to monitor all transactions. Second, many children are given antimalarials inappropriately when they present with a non-malarial febrile illness. In 2013, malaria-positive children consumed only 49% of the estimated 153 M (interquartile range (IQR): 140–167 M) private sector antimalarial courses purchased in African countries to treat under-five cases of fever.^13^ Disease progression due to improper (or lack of) diagnosis makes it harder to identify treatment failures caused by poor-quality antimalarials.

Although the private sector accounts for the majority of available antimalarial quality data, evidence indicates that drug quality is a problem in public and nongovernmental organization (NGO) sector distribution chains as well.^14^ Recently, falsified artemether–lumefantrine with labels mimicking those on the Global Fund/Affordable Medicines Facility-malaria's (AMFm's) quality-approved ACTs have been found to be widely distributed.^15^

Consumption of poor-quality antimalarials is associated with adverse health effects due to untreated malaria or toxic ingredients, accelerated emergence of drug-resistant malaria parasites because of subtherapeutic drug levels, and reduced consumer confidence in medicine, health care providers, and regulatory agencies.^16^–^18^ To date, there has been limited analysis of the burden caused by poor-quality antimalarials, and even the order of magnitude of health consequences remains to be investigated.

We used a simple uncertainty model to estimate the number of under-five malaria deaths attributable to poor-quality antimalarials in sub-Saharan Africa in 2013. Our approach was favored over more complex alternatives because of data limitations. This exercise is intended to aid researchers and policymakers by providing an approximate quantification of the burden of poor-quality antimalarials and guiding the collection of data needed to refine these estimates.

## Methods

We calculated the number of under-five deaths caused by *Plasmodium falciparum* (referred to as malaria) infections that persist because children consume poor-quality antimalarials instead of efficacious antimalarials across 39 countries in sub-Saharan Africa. We performed an uncertainty analyses using Latin hypercube sampling (LHS) (10,000 simulations) described by Blower and others.^19^ We performed all analyses using the R programming language and created an interactive, publicly available R package that can be used to perform and visualize our calculations using alternative input values (S1).

For each country, we calculated the number of under-five deaths caused by malaria treatment failure because of consumption of poor-quality antimalarials as the product: the number of private sector antimalarials consumed by malaria-positive children in 2013, the proportion of private sector antimalarials consumed that were poor-quality, and the case fatality rate (CFR) of under-five malaria-positive children who consumed poor-quality antimalarials. We included all countries for which antimalarial consumption estimates were available.^13^

LHS is an efficient, stratified Monte Carlo sampling design. Under the LHS scheme, a probability distribution is constructed for each input parameter that is not known with certainty. Supplemental Tables 1–3 describe the distributions used for the 79 input parameters in our model. These 79 parameters were categorized into the three input types, described in more detail below.

### Prevalence of poor-quality antimalarials.

We used available sample data describing the proportion of ACTs and sulfadoxine–pyrimethamine (SP) drugs failing chemical testing for nine countries (Cameroon, Ethiopia, Ghana, Kenya, Madagascar, Nigeria, Senegal, Tanzania, and Uganda) to construct probability distributions for the proportion of private sector sales that are poor-quality antimalarials.^20^,^21^ For each of these countries we used a normal approximation of the sampling distribution of the sample proportion. For the remaining 30 countries, we constructed a uniform distribution with a minimum (min) of 0% and a maximum (max) of 40%, consistent with the range of samples that failed testing reported in reviews of antimalarial quality surveys (Supplemental Table 1).^3^,^9^

### Antimalarial drug sales.

Cohen and others^13^ provided country-specific estimates of the number of private sector antimalarial sales for treating under-five malaria-positive children in 2013. For the LHS analysis, we constructed normal distributions for each nation (Supplemental Table 2). The methods for the sales estimates are described in detail elsewhere, but here we provide a brief overview.^13^ Household survey data were used to estimate the annual number of under-five fevers, the fraction of fevers treated with an antimalarial, and the fraction of fevers treated in the private sector in 2013. These values were multiplied to get the private sector under-five demand for antimalarials. Next, the fraction of fevers caused by malaria was calculated as a function of the *P. falciparum* parasite rate. This fraction was applied to the demand estimates to calculate the annual number of under-five malaria episodes treated with antimalarials purchased in the private sector.

### CFR of under-five malaria episodes treated with poor-quality antimalarials.

We constructed a uniform probability distribution around the mean CFR for under-five malaria cases used by WHO for constructing mortality estimates for low-transmission countries in Africa (min CFR of 0.2% and max CFR of 0.6%).^22^ This distribution was applied to all countries (Supplemental Table 3).

## Results

**Figure 1.**
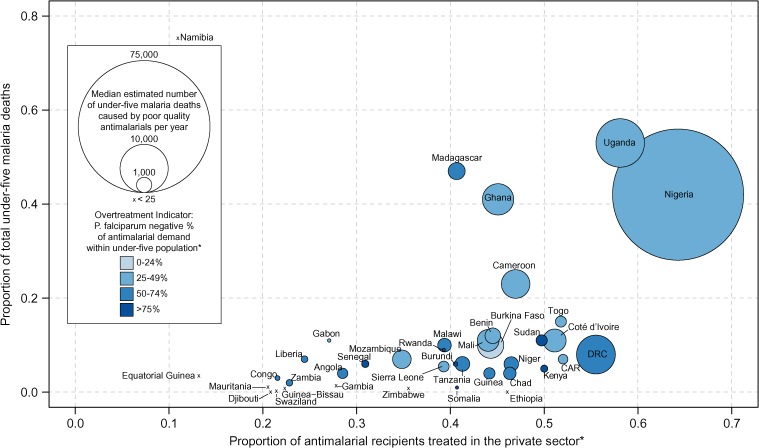
Estimated annual under-five malaria deaths due to treatment with poor-quality antimalarials. The median (of 10,000 samples) number of under-five malaria deaths caused by treatment with poor-quality antimalarials (radius) is plotted for 39 sub-Saharan countries for which antimalarial sales data were available. The y-axis depicts the poor-quality-antimalarial death estimates as a proportion of total 2010 under-five malaria death estimates made by World Health Organization (WHO) (Supplemental Table 4). The x-axis depicts the fraction of antimalarial recipients who are treated in the private sector. The color code depicts the proportion of private sector under-five antimalarial demand made by malaria-positive children. Darker shades reflect higher levels of overtreatment (i.e., febrile disease is presumptively treated as malaria). * Data were obtained from Cohen and others.^13^

**Figure 2.**
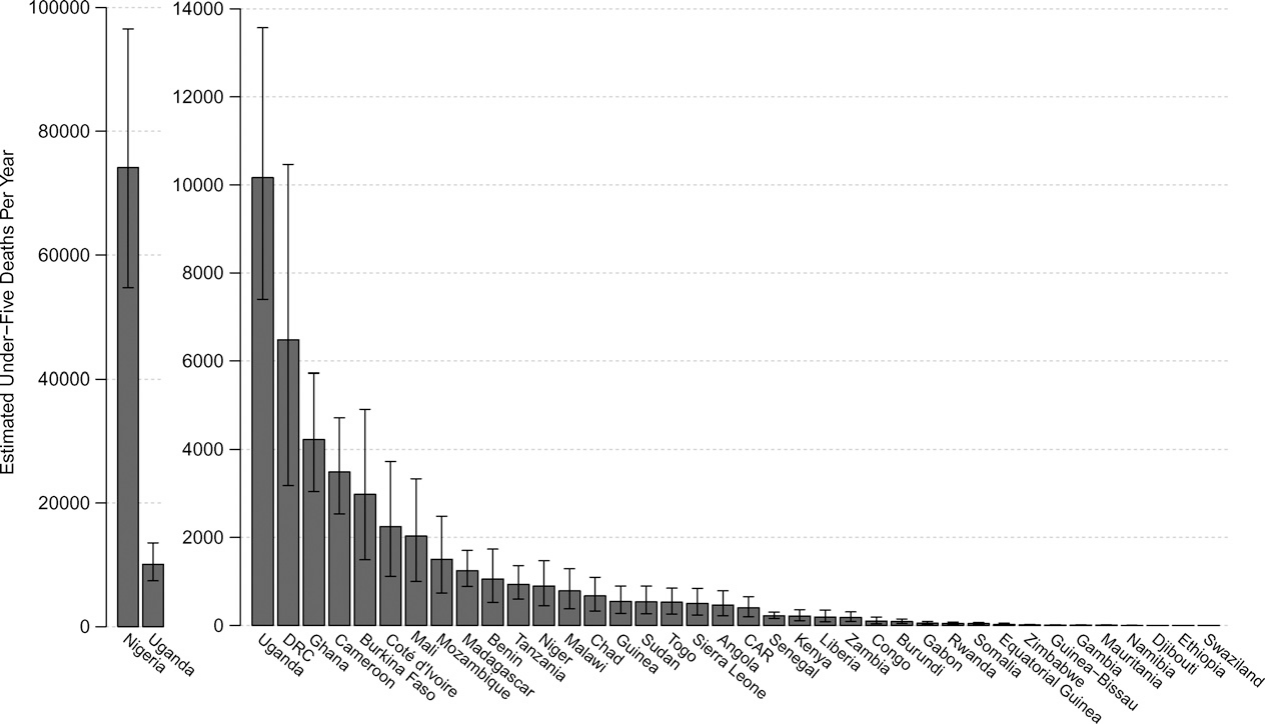
Estimated annual under-five malaria deaths due to treatment with poor-quality antimalarials. The median (derived from 10,000 simulations) number of under-five malaria deaths due to treatment with poor-quality antimalarials is plotted for 39 sub-Saharan countries. The error bars depict the interquartile range. Nigeria is plotted on a separate scale (Uganda is plotted twice for comparison).

**Figure 3.**
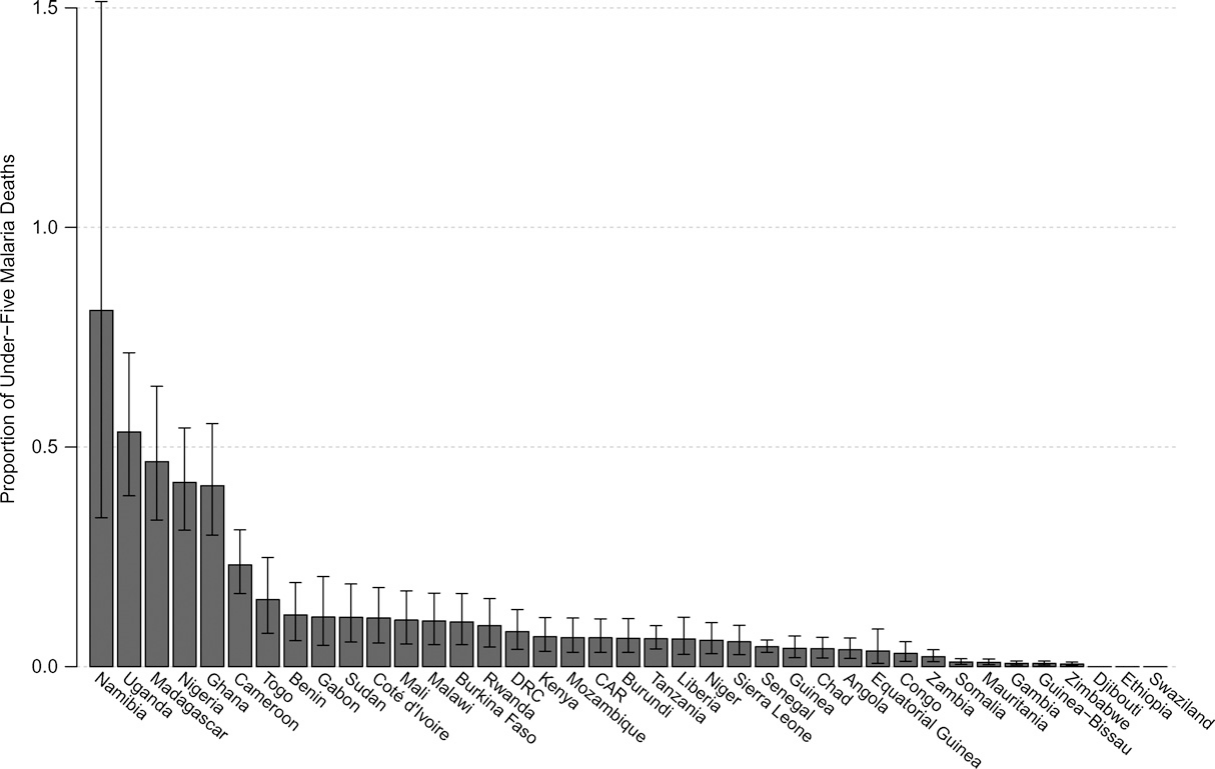
Deaths due to treatment with poor-quality antimalarials as proportion of under-five malaria deaths. The median (derived from 10,000 simulations) number of 2013 under-five malaria deaths due to treatment with poor-quality antimalarials is plotted for 39 sub-Saharan countries as a proportion of 2010 World Health Organization (WHO) under-five malaria death estimates (Supplemental Table 4). The error bars depict the interquartile range.

**Figure 4.**
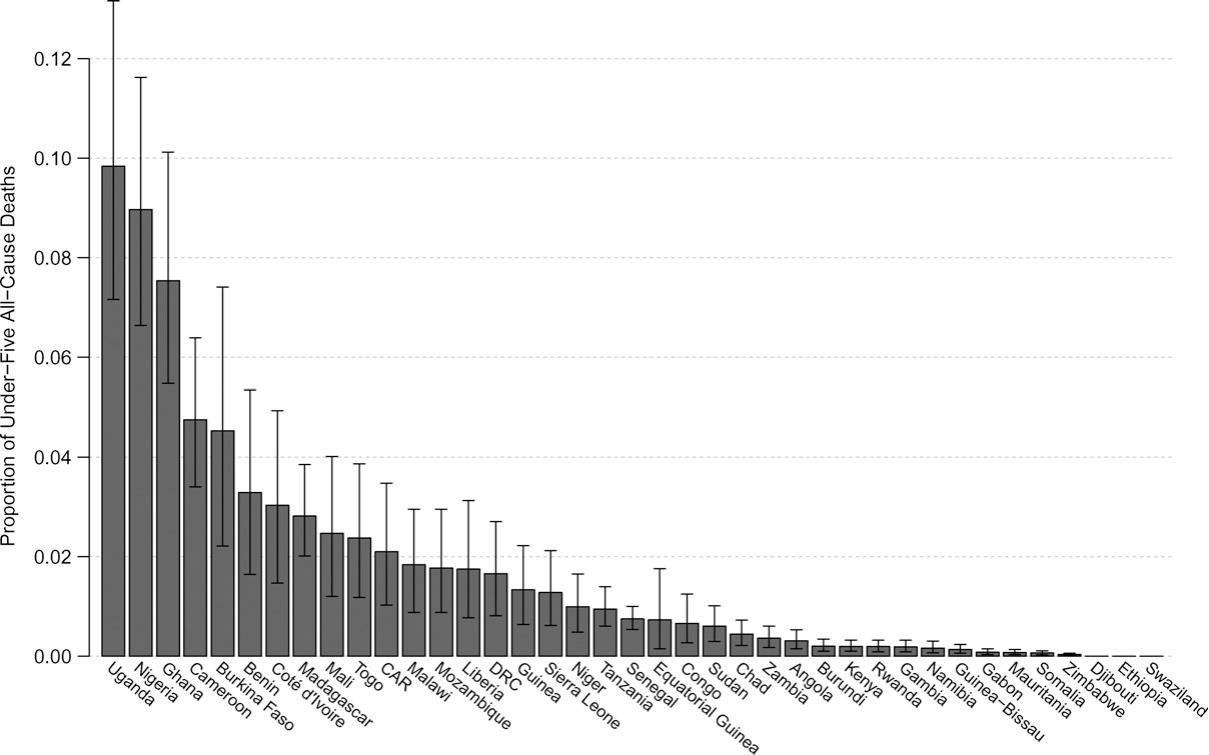
Under-five deaths due to treatment with poor-quality antimalarials as proportion of all-cause deaths. The median (derived from 10,000 simulations) number of 2013 under-five malaria deaths due to treatment with poor-quality antimalarials is plotted for 39 sub-Saharan countries as a proportion of 2012 World Health Organization (WHO) under-five all-cause death estimates (Supplemental Table 5). The error bars depict the interquartile range.

Our uncertainty analysis results provide estimates of under-five malaria mortality associated with consumption of poor-quality antimalarials. Table 1 presents the results from 10,000 calculations performed following the LHS scheme with the probability distributions described in Supplemental Tables 1–3. The estimated median number of under-five malaria deaths associated with consumption of poor-quality antimalarials across the 39 countries was 122,350 (IQR: 91,577–154,736). Nigeria, which had the largest estimated number of antimalarial sales to under-five malaria-positive children (30,225,237 courses), as well as the highest prevalence of poor-quality antimalarials (64%), accounted for a majority of the estimated deaths, with a median of 74,188 (IQR: 54,931–96,132) (Figures 1 and 2).^13^,^21^ Table 1 and Figure 3 present the number of deaths caused by poor-quality antimalarials as a percentage of 2010 under-five malaria death estimates. Figure 4 presents the number of deaths caused by poor-quality antimalarials as a proportion of 2012 all-cause under-five death estimates (Supplemental Table 5). Supplemental Table 4 presents the median number of deaths caused by poor-quality antimalarials alongside 2010 under-five deaths due to other causes.

## Discussion

There are several reasons it is difficult to estimate the health burden associated with widespread use of poor-quality antimalarials in sub-Saharan Africa. These reasons contribute to the uncertainty of our numerical results.

First, many poor-quality antimalarials are consumed by malaria-negative individuals. Our analysis attempts to reconcile the extensive use of poor-quality antimalarials with the significant overtreatment of febrile illness as malaria. Since many malaria-negative patients take antimalarials, improving febrile illness diagnostics might save more lives than reducing the prevalence of poor-quality antimalarials. Our analysis and methodology aim to inform this policy decision by isolating deaths caused by untreated malaria because of consumption of poor-quality antimalarials from deaths caused by untreated febrile illnesses because of diagnostic failures. Future research should focus on constructing estimates for the number of under-five deaths avertable through improved diagnosis of febrile illnesses.

Another difficulty with calculating the burden of poor-quality antimalarials is that not all legitimate antimalarials save lives. Even if poor-quality antimalarials are eliminated, there will still be excess mortality among children who consume genuine antimalarials (such as chloroquine) that are ineffective because of drug resistance. Because the “consequences” of poor-quality antimalarials depend on the efficacy of the genuine medicines, our estimates represent “the number of under-five deaths avoidable if children consume ACTs (or an effective alternative) instead of poor-quality antimalarials,” as opposed to “the number of under-five deaths avoidable if poor-quality antimalarials are eliminated.”

A further challenge is accurately selecting a CFR for under-five malaria episodes that are treated with poor-quality antimalarials. This is an important decision, since the imprecision of our total death estimates is directly tied to the variation in CFR inputs used across the 10,000 simulations. This process is complicated because the chemical composition of poor-quality antimalarials is variable.^8^ The major concerns for parameterizing the CFR are the antimalarial potencies of the poor-quality medicines and the presence of toxic ingredients. The CFR for a patient treated with a drug that contains toxins or banned pharmaceuticals exceeds the CFR of untreated malaria. When poor-quality antimalarials contain subtherapeutic levels of active pharmaceutical ingredients, the CFR should be lower than the rate of untreated malaria because the drugs might still prevent deaths due to malaria. When poor-quality antimalarials have no antimalarial properties and no toxic activity, the CFR should be identical to untreated malaria.

Selecting a CFR is further complicated by substantial disagreement regarding the CFR for untreated malaria.^23^ The median CFR for untreated under-five malaria from a Delphi survey of 22 malaria experts was 21.9% (219 deaths in 1,000 cases) for areas with low malaria transmission and 7.8% (78 deaths in 1,000 cases) for areas with high malaria transmission.^23^

The CFR range used for our estimates, 0.2–0.6% (2–6 deaths in 1,000 cases), is based on the 0.45% CFR (4.5 deaths in 1,000 cases) for under-five malaria cases (treated and untreated lumped together) used by WHO for constructing their malaria mortality estimates for low-transmission African countries.^22^ The WHO does not document how this 0.45% CFR is derived, but notes that “case fatality rates from malaria populations are not well documented … the case fatality rates assumed for different countries were assigned without regard to the availability and utilization of treatment for malaria, and in practice could vary from the rate used.”^24^

The selection of a CFR introduces a large uncertainty into our modeling framework. When compared with the results from the Delphi survey, our CFR range reflects a conservative assumption that poor-quality antimalarials are not entirely ineffective. This rate was set equal for all 39 countries because we lacked evidence to make country-specific estimates. With better data regarding the poor-quality antimalarials, we could update our estimates to accurately reflect the relative quantities of toxic, somewhat effective, and inert medication.

Despite the conservative CFR approach, we may be overestimating the number of deaths attributable to poor-quality antimalarials. Five countries had median poor-quality antimalarial death estimates that accounted for more than 40% of 2010 total under-five malaria-related deaths (Figure 2). Our uncertainty analysis illustrates the need for more confident assessments of input parameters and under-five malaria mortality estimates. Here we have used sales data from Cohen and others,^13^ where each “sale” corresponded to one under-five malaria episode that was treated with at least one antimalarial purchased in the private sector. Since Cohen and others^13^ acknowledged only a binary outcome for treatment-seeking behavior (whether the child received an antimalarial purchased in the private sector), the data did not tell us when children received multiple drugs for a single episode of malaria. In other words, our model did not support the scenario where a child who receives a poor-quality antimalarial also receives a quality antimalarial that can clear the infection. It would be possible to adjust the survey estimates if we had data describing the number of antimalarial courses consumed by one child who presents with a malaria episode in sub-Saharan Africa. This issue could be avoided if actual retail sales data were available instead of estimates derived from self-reported surveys. Furthermore, our analysis was not stratified by severity of disease. Severity could be an important determinant of the burden attributable to poor quality antimalarials. We may be overestimating deaths if severe malaria cases tend to be treated appropriately with quality drugs while uncomplicated cases receive poor-quality antimalarials.

Consumer awareness is another factor that complicates estimates of the burden of poor-quality antimalarials. For our estimates, we assumed that patients cannot distinguish between good- and poor-quality antimalarials so that the proportion of private sector antimalarials that are poor-quality is the same as the proportion of private sector antimalarials consumed that are poor quality. However, if consumers are able to avoid purchasing poor-quality antimalarials, then the prevalence of poor-quality antimalarials as determined by random samples would overestimate true consumption patterns.

Another obstacle with constructing death estimates is that the prevalence of poor-quality antimalarials is still widely unknown. Where the prevalence of poor-quality antimalarials is likely to be high, data are inherently scarce. For this reason we constructed country-specific probability distributions only for countries with available and reliable data (nine countries received country-specific probability distributions for the prevalence of poor-quality antimalarials; Supplemental Table 1). Even then, these reports may not accurately reflect the status of poor-quality antimalarials, since the samples were taken from a select number of private outlets and concerned antimalarials manufactured for all ages, rather than those used for under 5-year olds. Reports have consistently indicated high levels of poor-quality antimalarials across sub-Saharan Africa, but researchers have warned against generalizing the findings because of high variability.^3^,^8^–^10^,^18^ The insufficient sample sizes, convenience-based sampling designs, narrow sampling focuses (often examining non-artemisinin derivatives only), and lack of standardized chemical analysis techniques make it difficult to accurately estimate the prevalence and burden of poor-quality antimalarials.^10^ Recent projects undertaken by WWARN and ACTwatch have focused on accurate data collection and reporting.^25^,^26^ Readers can calculate and visualize alternative estimates using their own input settings by downloading the R package we developed for this research (S1).

A further limitation of this study is that our estimates quantify only one aspect of the burden of poor-quality antimalarials, that is, inadequate treatment of children with malaria. Other consequences include additional health concerns because of toxic ingredients and the substantial economic costs borne by families and health-care systems because of untreated malaria. Moreover, antimalarials that contain subtherapeutic levels of active ingredients can increase selection for drug-resistant parasites.^17^ Falsified medicines containing no antimalarial activity will not on their own engender resistance but will increase the risk of hyperparasitemia, recrudescence, and gametocytemia; these conditions could facilitate the spread of resistance.^8^,^27^ The loss of effective antimalarials to resistance is a serious concern that should be investigated closely.

Despite the previously discussed limitations of our analysis, this study is a first step toward accurately describing the burden of poor-quality antimalarials in sub-Saharan Africa. An estimated 122,350 (IQR: 91,577–154,736) under-five deaths were associated with consumption of poor-quality antimalarials in 39 sub-Saharan African countries in 2013. Although these results are only approximations, they suggest that poor-quality antimalarials are important contributors to under-five mortality.

According to our analysis the burden of private sector poor-quality antimalarials is concentrated in three countries (Nigeria, Uganda, and Democratic Republic of Congo), which account for 76.7% of all deaths (median estimates) associated with this problem. Therefore, international efforts to improve regulation, supply chains, and drug quality testing could focus on these areas.

Better data are needed both on the market prevalence of poor-quality antimalarials and on production origin. Chinese and Indian manufacturers are the most frequently cited sources of poor-quality antimalarials, but manufacturers in other countries may be involved as well.^18^ International flows of pharmaceuticals around the world should be tracked, particularly into sub-Saharan Africa, where the capacity for drug quality testing and patient protection is least developed.

The falsification of antimalarial pharmaceuticals is a multibillion-dollar business that is escalating because some countries lack appropriate deterrents.^28^ This problem requires investigations, prosecutions, and reductions of profit incentives (achievable by improving access to affordable, quality-assured antimalarials).^29^ Issues of poor quality control can also be targeted through stricter manufacturing standards (enforced by medicine regulatory authorities) or support systems that result in improved factory processes.^29^ Therefore, strengthening institutions in sub-Saharan Africa and in manufacturing countries such as India and China would be an effective and potentially cost-effective approach to meeting global goals for child survival.

## Supplementary Material

Supplemental Information.

## Figures and Tables

**Table 1:** Estimated under-five deaths caused by poor-quality antimalarials

Country	Min	First quartile	Median	Mean	Third quartile	Max	Median as % of malaria deaths*
All countries	38,772	91,577	122,350	125,012	154,736	269,705	22.33
Nigeria	13,220	54,931	74,188	77,231	96,132	206,618	41.96
Uganda	1,589	7,374	10,138	10,811	13,556	31,419	53.45
DRC	1	3,178	6,501	7,399	10,596	32,251	7.99
Ghana	390	3,068	4,223	4,527	5,669	14,439	41.22
Cameroon	309	2,521	3,520	3,756	4,730	12,119	23.18
Burkina Faso	0	1,463	2,991	3,421	4,892	15,146	10.18
Coté d'Ivoire	0	1,102	2,277	2,591	3,703	10,317	11.08
Mali	0	998	2,050	2,325	3,332	10,414	10.59
Mozambique	0	738	1,491	1,746	2,484	7,489	6.63
Madagascar	0	886	1,240	1,339	1,695	4,314	46.69
Benin	0	528	1,053	1,207	1,713	5,368	11.76
Niger	0	440	904	1,048	1,507	4,556	6.34
Tanzania	0	596	933	1,038	1,373	4,431	5.99
Malawi	0	379	792	899	1,272	4,317	10.42
Chad	0	326	670	766	1,089	3,198	4.10
Guinea	0	262	547	637	911	2,856	4.19
Sudan	0	268	539	626	901	2,644	11.24
Togo	0	260	523	598	851	2,733	15.27
Sierra Leone	0	241	499	582	829	2,754	5.65
Angola	0	224	464	552	788	2,753	3.83
CAR	0	196	399	460	661	1,860	6.55
Kenya	0	109	215	249	353	1,462	4.53
Liberia	0	84	192	242	344	1,319	6.81
Senegal	0	162	225	238	301	739	6.28
Zambia	0	88	181	215	304	1,072	2.28
Congo	0	40	99	130	187	865	3.00
Burundi	0	43	87	102	148	507	6.42
Gabon	0	21	51	65	92	407	11.26
Rwanda	0	22	46	53	77	248	9.20
Somalia	0	20	43	50	72	234	1.06
Equatorial Guinea	0	4	22	35	52	341	3.57
Zimbabwe	0	6	14	16	23	79	0.60
Guinea-Bissau	0	5	10	13	18	62	0.66
Gambia	0	4	9	11	16	61	0.73
Mauritania	0	3	8	10	14	71	0.94
Namibia	0	1	3	4	6	25	75.00
Swaziland	0	0	0	0	0	0	0.00
Djibouti	0	0	0	0	0	0	0.00
Ethiopia	0	0	0	0	0	0	0.00

CAR = Central African Republic; DRC = Democratic Republic of Congo.

This table displays the results from 10,000 simulations run following the Latin hypercube sampling scheme discussed in the methodology. The values presented depict the estimated number of under-five malaria deaths caused by treatment with poor-quality antimalarials for 39 sub-Saharan countries. The countries are listed in descending order according to the medians of 10,000 simulations (with the cumulative nation totals in the top row).

* 2010 WHO estimates of under-five malaria deaths (Supplemental Table 4).
