# Supplementary material for: Estimated Under-Five Deaths Associated with Poor-Quality Antimalarials in Sub-Saharan Africa
Source: Am J Trop Med Hyg. 2015 Jun 3;92(Suppl 6):119–26. doi: 10.4269/ajtmh.14-0725 (PMC4455082; doi:10.4269/ajtmh.14-0725)
Supplement: Supplementary file 1 [file SD7.pdf]

SUPPLEMENTAL INFORMATION

S1. THE “PQANTIMALARIALS” R PACKAGE

To complement this manuscript, we have created an interactive web tool that can be run locally on any computer that has R[1] by installing the “pqantimalarials” R package.[2] The tool allows users to select their own inputs and reproduce our calculations, so that our estimates can be refined as drug quality data improve. Below are the instructions for how to install and run our R package.

To install and load, enter the following lines into your R console:

```
install.packages(“pqantimalarials”)
library(pqantimalarials)
```

After installation run the web tool by typing:

```
webtool()
```

SUPPLEMENTAL REFERENCES

1. Team RDC, 2013. *R: A Language and Environment for Statistical Computing*. Vienna, Austria: R Foundation for Statistical Computing. Available at: http://www.R-project.org.
2. Patrick Renschler J, 2014. pqantimalarials. Available at: https://github.com/renschler/pqantimalarials. Accessed February 10, 2014.
3. Nayyar GM, Breman JG, Newton PN, Herrington J, 2012. Poor-quality antimalarial drugs in southeast Asia and sub-Saharan Africa. *Lancet Infect Dis 12:* 488–496.
4. World Health Organization, 2011. *Survey of the Quality of Selected Antimalarial Medicines Circulating in Six Countries of Sub-Saharan Africa*. Available at: http://www.who.int/medicines/publications/WHO_QAMSA_report.pdf. Accessed October 3, 2013.
5. U.S. Pharmacopeia, 2009. *Survey of the Quality of Selected Antimalarial Medicines Circulating in Madagascar, Senegal, and Uganda*. Available at: http://apps.who.int/medicinedocs/documents/s17069e/s17069e.pdf. Accessed October 3, 2013.
6. Cohen JM, Woolsey AM, Sabot OJ, Gething PW, Tatem AJ, Moonen B, 2012. Optimizing investments in malaria treatment and diagnosis. *Science 338:* 612–614.
7. Cairns M, Roca-Feltrer A, Garske T, Wilson AL, Diallo D, Milligan PJ, Ghani AC, Greenwood BM, 2012. Estimating the potential public health impact of seasonal malaria chemoprevention in African children. *Nat Commun 3:* 881.
8. Lubell Y, Staedke SG, Greenwood BM, Kamya MR, Molyneux M, Newton PN, Reyburn H, Snow RW, D’Alessandro U, English M, Day N, Kremsner P, Dondorp A, Mbacham W, Dorsey G, Owusu-Agyei S, Maitland K, Krishna S, Newton C, Pasvol G, Taylor T, von Seidlein L, White NJ, Binka F, Mills A, Whitty CJM, 2011. Likely health outcomes for untreated acute febrile illness in the tropics in decision and economic models; a Delphi survey. *PLoS ONE 6:* e17439.
9. World Health Organization, 2010. *Mortality: By Age Group and Cause: Children Aged 0 to 4 Years 2010*. Global Health Observatory Data Repository. Available at: http://apps.who.int/gho/data/node.main.GBDC-YEARS0-4?lang=en. Accessed March 13, 2014.
10. World Health Organization, 2014. *MDG 4: Child Health: Under-five Mortality by Country*. Available at: http://apps.who.int/gho/data/node.main.525. Accessed November 20, 2013.

Supplemental Table 1

Probability distributions for the prevalence of poor-quality antimalarials

Country	Distribution shape	Min	Mean	Max	Standard deviation	Reference no.
Angola	Uniform	0.0	0.2	0.4	NA	3
Benin	Uniform	0.0	0.2	0.4	NA	3
Burkina Faso	Uniform	0.0	0.2	0.4	NA	3
Burundi	Uniform	0.0	0.2	0.4	NA	3
Cameroon	Normal	NA	0.3659	NA	0.0752	4
CAR	Uniform	0.0	0.2	0.4	NA	3
Chad	Uniform	0.0	0.2	0.4	NA	3
Congo	Uniform	0.0	0.2	0.4	NA	3
Coté d'Ivoire	Uniform	0.0	0.2	0.4	NA	3
Djibouti	Uniform	0.0	0.2	0.4	NA	3
DRC	Uniform	0.0	0.2	0.4	NA	3
Equatorial Guinea	Uniform	0.0	0.2	0.4	NA	3
Ethiopia	Normal	NA	0.0000	NA	0.0000	4
Gabon	Uniform	0.0	0.2	0.4	NA	3
Gambia	Uniform	0.0	0.2	0.4	NA	3
Ghana	Normal	NA	0.3947	NA	0.0793	4
Guinea	Uniform	0.0	0.2	0.4	NA	3
Guinea-Bissau	Uniform	0.0	0.2	0.4	NA	3
Kenya	Normal	NA	0.0465	NA	0.0321	4
Liberia	Uniform	0.0	0.2	0.4	NA	3
Madagascar	Normal	NA	0.3019	NA	0.0631	5
Malawi	Uniform	0.0	0.2	0.4	NA	3
Mali	Uniform	0.0	0.2	0.4	NA	3
Mauritania	Uniform	0.0	0.2	0.4	NA	3
Mozambique	Uniform	0.0	0.2	0.4	NA	3
Namibia	Uniform	0.0	0.2	0.4	NA	3
Niger	Uniform	0.0	0.2	0.4	NA	3
Nigeria	Normal	NA	0.6393	NA	0.0615	4
Rwanda	Uniform	0.0	0.2	0.4	NA	3
Senegal	Normal	NA	0.4355	NA	0.0630	5
Sierra Leone	Uniform	0.0	0.2	0.4	NA	3
Somalia	Uniform	0.0	0.2	0.4	NA	3
Sudan	Uniform	0.0	0.2	0.4	NA	3
Swaziland	Uniform	0.0	0.2	0.4	NA	3
Tanzania	Normal	NA	0.1111	NA	0.0468	4
Togo	Uniform	0.0	0.2	0.4	NA	3
Uganda	Normal	NA	0.2561	NA	0.0482	5
Zambia	Uniform	0.0	0.2	0.4	NA	3
Zimbabwe	Uniform	0.0	0.2	0.4	NA	3

CAR = Central African Republic; DRC = Democratic Republic of Congo.

Under the Latin hypercube sampling scheme, a probability distribution is constructed for each input parameter that is not known with certainty. Supplemental Table 1 lists the distributions used for the 39 country-specific estimates of the proportion of private sector antimalarial sales that are of poor quality. For nine nations (Cameroon, Ethiopia, Ghana, Kenya, Madagascar, Nigeria, Senegal, Tanzania, and Uganda) we constructed a normal distribution using available drug quality survey data with mean = sample proportion (p) and variance = p × (1 − p)/sample size.

Supplemental Table 2

Probability distributions for antimalarial drug sales to *Plasmodium falciparum* positive under-five children

Country	Distribution shape	Mean	Standard deviation*
Angola	Normal	691,292	209,651
Benin	Normal	1,513,257	319,498
Burkina Faso	Normal	4,285,803	913,214
Burundi	Normal	128,086	32,682
Cameroon	Normal	2,568,023	594,934
CAR	Normal	577,328	130,810
Chad	Normal	956,155	214,179
Congo	Normal	163,577	86,732
Coté d'Ivoire	Normal	3,224,374	706,333
Djibouti	Normal	31	17
DRC	Normal	9,250,372	2,127,573
Equatorial Guinea	Normal	41,745	39,776
Ethiopia	Normal	126,446	36,729
Gabon	Normal	80,957	36,753
Gambia	Normal	14,145	4,209
Ghana	Normal	2,871,689	637,899
Guinea	Normal	795,914	198,983
Guinea-Bissau	Normal	16,429	5,756
Kenya	Normal	1,317,873	290,113
Liberia	Normal	303,031	131,343
Madagascar	Normal	1,109,482	289,818
Malawi	Normal	1,128,586	261,895
Mali	Normal	2,913,747	630,773
Mauritania	Normal	12,857	4,700
Mozambique	Normal	2,181,412	548,964
Namibia	Normal	5,299	2,576
Niger	Normal	1,303,059	301,260
Nigeria	Normal	30,225,236	6,108,047
Rwanda	Normal	67,444	16,421
Senegal	Normal	137,347	35,679
Sierra Leone	Normal	730,160	199,490
Somalia	Normal	63,210	16,972
Sudan	Normal	782,510	191,311
Swaziland	Normal	5	2
Tanzania	Normal	2,329,826	522,977
Togo	Normal	748,210	158,948
Uganda	Normal	10,560,843	2,310,272
Zambia	Normal	270,082	77,633
Zimbabwe	Normal	21,013	6,155

CAR = Central African Republic; DRC = Democratic Republic of Congo.

Under the Latin hypercube sampling scheme, a probability distribution is constructed for each input parameter that is not known with certainty. Supplemental Table 3 displays the estimates of 2013 antimalarial sales to *P. falciparum* positive under-five children in 39 sub-Saharan nations obtained from Cohen and others.[6] These data were used to construct country-specific normal probability distributions.

*The data received from Cohen and others[6] contained median and quartile estimates only. The standard deviation was inferred from the interquartile range assuming a normal distribution.

Supplemental Table 3

Probability distribution for case fatality rate of under-five malaria episodes treated with poor-quality antimalarials

Country	Distribution shape	Min	Mean	Max	Reference no.
All countries	Uniform	0.002	0.004	0.006	7,8

Under the Latin hypercube sampling scheme, a probability distribution is constructed for each input parameter that is not known with certainty. Supplemental Table 3 lists the distribution used for the case fatality rate for malaria infections in under-five children who were treated with poor-quality antimalarials.

Supplemental Table 4

Under-five deaths by cause

Country	Poor-quality antimalarials	Malaria	Measles	HIV/AIDS	Acute lower respiratory infections	Birth asphyxia and birth trauma
Angola	464	12,128	22	2,160	20,897	9,926
Benin	1,053	8,957	124	344	6,744	3,330
Burkina Faso	2,991	29,368	3,541	848	21,764	7,883
Burundi	87	1,355	11	2,296	7,294	3,388
Cameroon	3,520	15,183	9	4,871	14,364	7,251
CAR	399	6,087	52	729	3,756	1,824
Chad	670	16,356	27	2,492	14,956	5,912
Congo	99	3,303	49	617	1,841	1,205
Coté d'Ivoire	2,277	20,554	385	2,570	11,800	7,752
Djibouti	0	13	24	98	445	232
DRC	6,501	81,400	640	6,051	87,007	34,955
Equatorial Guinea	22	617	3	228	396	265
Ethiopia	0	4,514	10,450	5,481	57,844	27,509
Gabon	51	453	97	252	332	297
Gambia	9	1,238	20	176	949	625
Ghana	4,223	10,246	456	1,836	7,467	6,344
Guinea	547	13,041	15	603	7,885	4,278
Guinea-Bissau	10	1,505	40	232	1,457	681
Kenya	215	3,156	63	8,325	20,474	13,122
Liberia	192	3,058	1,626	361	2,383	1,527
Madagascar	1,240	2,656	644	124	8,161	5,096
Malawi	792	7,604	1,317	7,468	7,865	5,237
Mali	2,050	19,349	4,172	477	24,478	9,116
Mauritania	8	853	980	49	2,204	1,284
Mozambique	1,491	22,482	1,546	11,858	17,814	9,877
Namibia	3	4	89	339	294	268
Niger	904	15,085	273	722	21,691	7,052
Nigeria	74,188	176,793	9,728	31,742	143,596	73,781
Rwanda	46	500	3	779	4,782	3,177
Senegal	225	4,968	834	520	5,518	3,881
Sierra Leone	499	8,831	41	383	6,755	2,882
Somalia	43	4,063	99	395	15,256	5,541
Sudan	539	4,796	976	2,393	26,966	15,377
Swaziland	0	1	1	611	365	228
Tanzania	933	14,715	936	7,374	19,835	14,778
Togo	523	3,425	56	523	3,088	1,820
Uganda	10,138	18,966	30	10,355	24,078	11,878
Zambia	181	7,928	2,593	6,674	8,901	5,380
Zimbabwe	14	2,328	364	5,926	3,314	2,813

Country	Poor-quality antimalarials	Congenital anomalies	Diarrheal diseases	Injuries	Meningitis/encephalitis	Other communicable, perinatal, and nutritional conditions
Angola	464	8,375	18,598	4,322	3,043	17,911
Benin	1,053	1,743	3,958	1,348	457	6,333
Burkina Faso	2,991	5,123	14,648	3,762	2,485	19,635
Burundi	87	1,156	5,605	1,782	1,319	6,164
Cameroon	3,520	4,799	12,150	3,212	1,653	14,144
CAR	399	810	2,540	653	471	2,373
Chad	670	3,060	11,234	2,602	2,790	6,116
Congo	99	772	951	364	90	1,569
Coté d'Ivoire	2,277	3,165	6,991	2,201	1,004	9,138
Djibouti	0	187	243	90	20	461
DRC	6,501	20,476	59,533	14,209	6,936	79,264
Equatorial Guinea	22	134	205	67	22	331
Ethiopia	0	9,989	38,534	15,656	15,304	31,538
Gabon	51	244	203	86	20	209
Gambia	9	371	555	215	108	759
Ghana	4,223	3,910	4,143	2,214	929	6,521
Guinea	547	1,735	4,808	1,462	837	4,727
Guinea-Bissau	10	406	1,016	275	180	944
Kenya	215	7,117	11,461	6,650	2,763	19,962
Liberia	192	783	1,521	582	299	1,556
Madagascar	1,240	2,368	4,627	3,003	1,084	6,246
Malawi	792	2,150	4,025	2,235	864	7,617
Mali	2,050	5,907	17,977	3,861	2,544	17,819
Mauritania	8	853	1,458	511	229	1,993
Mozambique	1,491	3,836	10,696	3,304	1,683	13,305
Namibia	3	226	121	135	21	338
Niger	904	3,237	14,499	4,361	3,465	13,681
Nigeria	74,188	36,392	97,638	24,804	26,820	71,838

(continued)

Supplemental Table 4

Continued

Country	Poor-quality antimalarials	Congenital anomalies	Diarrheal diseases	Injuries	Meningitis/encephalitis	Other communicable, perinatal, and nutritional conditions
Rwanda	46	1,230	2,781	1,366	557	4,659
Senegal	225	2,684	2,975	1,489	570	3,959
Sierra Leone	499	1,925	4,821	1,236	907	4,961
Somalia	43	3,003	9,952	2,004	2,259	6,164
Sudan	539	7,176	17,420	7,261	6,185	17,870
Swaziland	0	109	184	126	44	420
Tanzania	933	6,127	11,391	6,722	2,911	17,695
Togo	523	1,110	1,966	733	327	2,715
Uganda	10,138	6,077	14,085	6,644	3,491	15,925
Zambia	181	2,406	5,720	2,300	1,434	7,261
Zimbabwe	14	1,350	2,263	1,032	366	3,183

Country	Poor-quality antimalarials	Other noncommunicable diseases	Pertussis	Prematurity	Sepsis and other infectious conditions of the newborn
Angola	464	183	1,292	15,255	6,978
Benin	1,053	157	176	4,556	815
Burkina Faso	2,991	246	374	11,332	2,333
Burundi	87	151	78	4,871	2,953
Cameroon	3,520	412	375	10,423	4,345
CAR	399	85	410	2,476	1,214
Chad	670	166	2,093	8,725	3,516
Congo	99	95	46	1,647	214
Coté d'Ivoire	2,277	188	391	10,421	4,209
Djibouti	0	19	10	368	30
DRC	6,501	1,709	4,355	47,888	20,160
Equatorial Guinea	22	13	97	386	132
Ethiopia	0	621	1,847	40,567	17,331
Gabon	51	19	134	489	144
Gambia	9	29	11	836	238
Ghana	4,223	280	212	8,951	3,385
Guinea	547	100	1,006	5,686	2,266
Guinea-Bissau	10	21	127	930	388
Kenya	215	1,015	991	18,344	8,685
Liberia	192	48	328	1,964	1,030
Madagascar	1,240	446	419	7,062	2,713
Malawi	792	531	159	7,436	2,723
Mali	2,050	168	351	13,734	4,115
Mauritania	8	60	92	1,836	857
Mozambique	1,491	601	537	13,068	5,120
Namibia	3	10	30	460	116
Niger	904	97	1,609	11,869	2,548
Nigeria	74,188	1,619	20,197	101,480	52,295
Rwanda	46	224	69	4,289	1,829
Senegal	225	181	194	5,253	1,676
Sierra Leone	499	59	366	3,953	1,980
Somalia	43	213	1,518	7,624	3,125
Sudan	539	551	620	22,485	10,655
Swaziland	0	10	10	381	121
Tanzania	933	791	764	20,403	9,028
Togo	523	84	78	2,592	858
Uganda	10,138	1,099	3,324	18,240	7,276
Zambia	181	393	278	8,059	3,492
Zimbabwe	14	237	274	4,028	1,866

AIDS = acquired immunodeficiency syndrome; CAR = Central African Republic; DRC = Democratic Republic of Congo; HIV = human immunodeficiency syndrome; WHO = World Health Organization.

Supplemental Table 4 presents the median estimated number of 2013 under-five malaria deaths associated with poor-quality antimalarials (calculated using the original inputs presented in Supplemental Tables 1, 2, and 3) alongside 2010 under-five death estimates because of other causes (malaria, measles, HIV/AIDS, acute lower respiratory infections, birth asphyxia/birth trauma, congenital anomalies, diarrheal diseases, injuries, meningitis/encephalitis, other communicable/perinatal/nutritional conditions, other noncommunicable diseases, pertussis, prematurity, and sepsis/other infectious conditions of the newborn). The 2010 under-five death estimates were taken from the WHO's Global Health Observatory Data Repository.[9]

Supplemental Table 5

All-cause under-five mortality

Country	2012 under-five death estimates (thousands)
Angola	148
Benin	32
Burkina Faso	66
Burundi	43
Cameroon	74
CAR	19
Chad	150
Congo	15
Coté d'Ivoire	75
DRC	391
Djibouti	2
Equatorial Guinea	3
Ethiopia	205
Gabon	62
Gambia	5
Ghana	56
Guinea	41
Guinea-Bissau	8
Kenya	108
Liberia	11
Madagascar	44
Malawi	43
Mali	83
Mauritania	11
Mozambique	84
Namibia	2
Niger	91
Nigeria	827
Rwanda	24
Senegal	30
Sierra Leone	39
Somalia	65
Sudan	89
Swaziland	3
Tanzania	98
Togo	22
Uganda	103
Zambia	50
Zimbabwe	39

CAR = Central African Republic; DRC = Democratic Republic of Congo.

Supplemental Table 5 presents 2012 all-cause under-five death estimates (in thousands) produced by the World Health Organization (WHO). The estimates were retrieved from the WHO's Global Health Observatory Data Repository.[10]
